# Adapting continuing medical education for post-conflict areas: assessment in Nagorno Karabagh - a qualitative study

**DOI:** 10.1186/1478-4491-12-39

**Published:** 2014-08-06

**Authors:** Arin A Balalian, Hambardzum Simonyan, Kim Hekimian, Byron Crape

**Affiliations:** 1 Fund for Armenian Relief of America (FAR), Healthcare Department, #22 Khorenatsi Street, Yerevan, Republic of Armenia; 2 Institute for Human Nutrition, Columbia University, 630 West 168th Street PH 15 East, Suite 1512, New York, NY 10032, USA; 3 School of Public Health, American University of Armenia, #40 Baghramyan Street, Yerevan, Republic of Armenia

**Keywords:** Post-conflict zones, Human resources in health care in post-conflict zones, Infrastructures in post-conflict zones, Qualitative evaluation, Nagorno Karabagh

## Abstract

**Background:**

One of the major challenges in the current century is the increasing number of post-conflict states where infrastructures are debilitated. The dysfunctional health care systems in post-conflict settings are putting the lives of the populations in these zones at increased risk. One of the approaches to improve such situations is to strengthen human resources by organizing training programmes to meet the special needs in post-conflict zones. Evaluations of these training programmes are essential to assure effectiveness and adaptation to the health service needs in these conditions.

**Methods:**

A specialized qualitative evaluation was conducted to assess and improve a post-conflict continuing medical education (CME) programme that was conducted in Nagorno Karabagh. Qualitative research guides were designed for this post-conflict zone that included focus group discussions with physician programme participants and semi-structured in-depth interviews with directors of hospitals and training supervisors.

**Results:**

Saturation was achieved among the three participating groups in the themes of impact of participation in the CME and obstacles to application of obtained skills. All respondents indicated that the continuing medical education programme created important physician networks absent in this post-conflict zone, updated professional skills, and improved professional confidence among participants. However, all respondents indicated that some skills gained were inapplicable in Nagorno Karabagh hospitals and clinics due to lack of appropriate medical equipment, qualified supporting human resources and facilities.

**Conclusion:**

The qualitative research methods evaluation highlighted the fact that the health care human resources training should be closely linked to appropriate technologies, supplies, facilities and human resources available in post-conflict zones and identified the central importance of creating health professional networks and professional confidence among physicians in these zones. The qualitative research approach most effectively identifies these limitations and strengths and can directly inform the optimal adjustments for effective CME planning in these difficult areas of greatest need.

## Background

Post-conflict zones are characterized by political uncertainty, civil unrest and a struggling economy [1]. Infrastructures, including health service infrastructures, are debilitated [2]. Post-conflict zones are widespread. Currently there are more post-conflict countries in Africa than those in conflict [3]. Many countries in Latin America [4,5] and South Asia [6] are in a state of post-conflict and other countries are currently transitioning into post-conflict states as a result of the ‘Arab Spring’ [7].

Several studies have found higher mortality rates after conflicts than during conflicts as a result of collapsed infrastructures and the deterioration of health care services [8-12]. To improve health conditions during post-conflict periods, it is essential to train, empower and support post-conflict health professionals to rebuild health services to an adequate level.

Continuing medical education (CME) strengthens professional clinical knowledge and skillsets of health care providers to assure competent delivery of improved health care services to the population [13]. However, standard CME programmes are designed for stable environments and not adapted for post-conflict zones where the need for CME is greatest [14,15]. Training of physicians is complicated in post-conflict zones, where health care infrastructures are poor, human resources are lacking and skills among health providers are inadequate [16]. Post-conflict zone health systems are chronically underfunded. They sorely lack in educational materials, modern equipment and supplies and health professional networks. Inadequate health care support systems, outdated protocols, the presence of informal payments and low morale among health professionals are common in post-conflict zones [16-20]. In these conditions, CME is the most cost-effective means of substantially improving health services and health service delivery in post-conflict zones, though the design of post-conflict CME has rarely been explored.

The critical need for effective training of physicians in these post-conflict zones requires different priorities and customized approaches in the design of CME that are especially adapted to these zones. Comprehensive assessment of trial CME in post-conflict zones allows adaptation of changes to future CME to meet the special needs found in these zones, assuring effective application, strengthened health services, and improved outcomes.

There are various methods to evaluate CME programmes in developed countries. Some include surveying patients on quality and satisfaction of the medical care they received by their attending physicians after the physicians received CME training [21]. Others audit charts of the physicians before and after attending CME training [22-24]. However, these approaches do not address many of the obstacles for optimizing CME specifically to post-conflict zones, such as physicians’ professional confidence, limitations on resources, and weak infrastructure [16,18,25].

Only a few studies have assessed the effectiveness of health care professionals’ training in post-conflict zones. An evaluation of a six-month primary health care training programme in Kosovo found that the programme was successful in refreshing physicians’ knowledge and improving professional confidence in their skills [26]. Another evaluation of an antenatal care training programme conducted in Kosovo concluded that although knowledge scores of physicians increased, such programmes must be offered regularly to sustain this gain [27]. An evaluation of a first aid course in Nagorno Karabagh (NK) found participants’ knowledge score was higher at the post-training test as compared to the baseline. However, three months following the course there was a substantial decline in knowledge score. The authors suggested that regular training sessions as well as application of knowledge gained would prevent the decline in knowledge [25]. None of these evaluations assessed the appropriateness of the training content in the context of the post-conflict zones where their sessions were conducted.

Utilizing qualitative methods for evaluation of first-time CME in post-conflict zones provides for the identification and characterization of infrastructural, structural and professional obstacles for effective training programmes specific to these zones [18,26,28]. Many of these obstacles are absent in stable non-conflict settings. Findings from qualitative evaluations of first-time post-conflict CME inform the adaptation of future CME to effectively address these obstacles - improving health care and outcomes. Qualitative methods have also been found to be more effective than quantitative methods in evaluating the contextual impact of institutional cultures on physicians [29].

NK is a mountainous region in the Southern Caucasus that was previously located in the Soviet Republic of Azerbaijan in the Soviet Union [18,30]. With the breakup of the Soviet Union looming, in 1988 NK declared itself independent of Azerbaijan, leading to a war involving Azerbaijan, Armenia and the habitants of NK [30].

At the time of the cease-fire in 1994, the health service infrastructure and the health care system in this post-conflict region had been severely compromised [18,25,31]. To date, there are no peace accords in place, with violence flaring up regularly along the border of NK and Azerbaijan. NK remains isolated, without international recognition as an independent state [30].

As a result, the quality of health care services and medical education has severely declined [18,25]. The region does not have the resources, the human resource capacity or the infrastructure to provide CME [25]. To alleviate this situation, Fund for Armenian Relief in collaboration with the Armenian American Health Professionals Organization, designed and conducted a hands-on CME training programme for physicians in NK. For one month, CME was conducted for NK physicians in Yerevan, Armenia by internationally-trained physicians with diverse specializations. The CME programme provided patient rounds and hands-on training to update professional medical knowledge and skills.

A standard quantitative survey with close-ended questions was conducted at the conclusion of the CME, the first CME offered for NK physicians. A follow-up qualitative evaluation of the CME was designed to address the issues that could not be addressed by the standard quantitative survey. The close-ended questions on the quantitative survey instrument were unable to identify and characterize the utilization of knowledge and skills gained from the CME when the physicians return to their workplaces. Neither did it adequately profile the breadth and depth of the barriers and obstacles of post-conflict CME effectiveness.

The overarching purpose of the qualitative evaluation was to characterize and identify obstacles and barriers to effective CME in a post-conflict zone, to direct the design of CME in these zones to improve health care services and health outcomes. The qualitative evaluation research questions included the following: 1. What was the professional impact of the training programme on the health care services in NK? 2. How well are skills and knowledge acquired by this training programme utilized in their home clinics and hospitals? 3. What are the barriers and obstacles in this context for effective application of the skills and knowledge acquired?

## Methods

The Institutional Review Board of American University of Armenia approved the study for adherence to internationally accepted ethical standards.

The investigators utilized qualitative data collection methods, which included semi-structured in-depth interviews (IDIs) and focus group discussions (FGDs), for the assessment of the first CME programme conducted in NK, a resource-poor post-conflict state. Focus group and IDI guides were developed and pretested prior to data collection. Three groups of informants participated in the study: NK physicians who were recipients of the CME programme, directors of hospitals in NK, and the CME training supervisors based in Yerevan.

The study team developed three different interview guides for focus group discussions conducted with the recipients of the CME programme, IDIs with the hospital directors and IDIs with the training supervisors respectively. In total, 40 respondents were recruited for the study. All the respondent groups gave oral consent to participate in the study.

FGDs: the researchers invited all 56 physicians who had participated in the CME programme to participate in the evaluation. Only one physician refused to participate in FGD. Two participants left the FGDs early due to conflicts with work schedules.

The physicians participating in FGDs had diverse professional specializations. The majority of FGD participants were female, with only two males participating. Twenty FGD participants were from Stepanakert, the capital city of NK, and the rest came from other communities.

IDIs with hospital directors in NK: the researchers conducted semi-structured IDIs with all accessible five hospital directors in NK who provided consent. Only two hospital directors in NK were not invited to be interviewed due to geographic inaccessibility. There were no refusals among hospital directors.

IDIs with CME trainings supervisors: the research team invited six training supervisors out of 41 to participate in IDIs based on the number of CME participants they supervised. Those who had largest numbers of trainees were invited for interviews using a purposive sampling scheme. There were no refusals among CME training supervisors who were invited to participate in IDIs.

After five FGDs with twenty-nine physicians and six IDIs with trainings supervisors were conducted, data collection was stopped. Consistent redundancy of information was observed among all three participant groups in the themes of 1) impact of participation in the CME, and 2) obstacles to application of obtained skills.

The data collected were transcribed and subsequently translated from Armenian to English by two translators before analysis. The study team conducted inductive content analysis to analyze the data. The transcribed data were coded and similar ideas were grouped and categorized subsequently under the same subcategories, which was followed by abstraction. The study team obtained information from heterogeneous groups and reported triangulated results within and between groups/interviews.

## Results

The main categories identified were the creation of professional networks, increased professional confidence in managing patients and obstacles to applying acquired knowledge and skills in their context. These three categories are further elaborated in the following sections.

### Creation of a professional network

Most of the programme participants and training supervisors noted that one of the major achievements of the CME programme was the creation and development of health care professional networks, previously lacking in this post-conflict zone. This was essential for rebuilding the health services infrastructure.

All CME programme supervisors who participated in IDIs consistently indicated that they maintained contact with programme participants to support and assist them. This assistance included providing consultations, referrals, and arrangements and information about upcoming conferences and training programmes:

*‘My connection with the physicians did not end after the training. We are not limiting ourselves to the programme; we continue to keep our contacts through phone calls. The connection is always present. Whenever they (the programme participants) need to consult they call us by phone; when oral consultation it is not enough, they send their patients here for consultation. So our relation is more close and tight.’* (CME supervisor)

Professional Network created by the programme is assisting CME participants in transferring patients to hospitals for more specialized care:

*‘I think using the telecommunication resources to be in contact with the physicians from Armenia is an effective approach which was not adequate before, for example, I am having my contact with my supervisor and other participants who participated in the programme with me. This helps me a lot when I want to transfer a patient to another hospital.’* (CME participant)

### Professional confidence

All three groups who participated in the evaluation (CME programme participants, training supervisors and hospital directors) agreed that professional confidence in treatment and management of patients among CME participants was substantially increased.

Hospital directors noted that the confidence of physicians in managing their patients had been enhanced by the CME programme:

‘*It is clearly noticed especially in physicians who have not left this region in the last ten years. The gynecologists have now been trained in new methods; the pediatricians were also very pleased. You can see this new confidence among physicians who have been recently trained.’* (NK hospital director)

Physicians whose practice were interrupted as a result of the war, returned to practice with an increased professional confidence provided by the training:

*‘I had not been a practicing physician for a long time but participating in these trainings refreshed and restored my knowledge. We received a new ultrasound machine for eyes, and nobody else knew how to use it. I used it with confidence, thanks to the training.’* (CME participant)

Increased professional confidence translated into other positive consequences. The CME training supervisors in Armenia noted that referral rates have declined with increased professional confidence found among NK physicians as a result of the training:

*‘Their knowledge is deepened and they are more confidently managing their patients now themselves than older times when they might have sent the same patients to Yerevan.’* (CME supervisor)

### Obstacles to application

Several programme participants reported effective use of some new skills acquired in their clinical settings. However, all three groups emphasized that the lack of appropriate facilities and equipment is an obstacle in applying many of the skills acquired in the CME training. A CME participant explained:

*‘I participated in numerous percutaneous coronary intervention procedures in the cardiology hospital in Yerevan; however we neither have the capacity in our hospital nor the equipment to perform those procedures.’* (CME participant)

Other physicians who participated in the CME programme also noted the lack of medical equipment and supplies in this post-conflict area was a barrier to application of the acquired skills:

*‘The training had a great impact on my professional development. I refreshed my knowledge and learned many new methods and approaches and new guidelines, but I cannot use most of them because of our poor resources. And the problem is if I don’t apply my skills then I will start to forget them.’* (CME participant)

The lack of appropriate medical supplies and equipment was also emphasized by directors of hospitals in NK:

*‘The problem is more about having the medical equipment in Nagorno Karabagh. For example, the programme participants go and learn many new methods in surgery such as laparoscopy. If they do not have the means to apply their knowledge, then it becomes a big problem. I think the most important barrier is our lack of technology.’* (NK hospital director)

Several physicians noted the lack of qualified supporting human resources, hospital staff not trained in the new methods, is also placing restrictions on applications of new modern methods in NK that were learned in the CME.

For example, when a laboratory analysis is necessary for diagnosis of a disease and the laboratory staff is not able to conduct it in NK due to the lack of supporting technical and human resources:

*‘The laboratory physicians are not conducting some laboratory analysis since they do not know how to; they need training.’* (CME participant)

## Discussion

CME is an effective means of strengthening post-conflict zone dysfunctional health care services [15]. However, standard CME is not designed for the special needs found in these zones. Qualitative evaluation methods are the most effective means to adapt CME to post-conflict zones.

The qualitative evaluation found that two central needs for effective CME in this post-conflict zone were to re-establish professional health care networks and professional confidence in management and treatment of the patients. This evaluation also identified the obstacles that restrict participants from applying their newly acquired skills in their health care settings in NK. None of these findings were identified by a previous quantitative survey designed to evaluate the CME programme. These findings can guide programme planners for future improved CME design for these conditions.

The creation and development of professional networks among physicians in NK, their training supervisors, and other colleagues was a central success of the CME programme. This was essential for the rebuilding of the infrastructure of the health care services in NK. The programme was also essential in strengthening the professional confidence among the physicians in management and treatment of their patients. Systematic reviews have found that professional confidence among physicians plays a significant role in appropriate management and treatment of the patients, following proper protocols and best practices [32,33]. Rebuilding the confidence among physicians working in post-conflict zone is one of the challenges of CME trainings [16,18,26,34].

The qualitative evaluation found shortcomings in the design of the CME. The CME trained the physicians using medical equipment and supplies that were lacking in NK health facilities. This major obstacle facing NK health facilities was a major concern from all three participating groups in the qualitative evaluation.

The lack of medical equipment, supplies, and facilities in this post-conflict zone contributed to a lack of practical application of physicians’ acquired knowledge and skills from the CME, leading to probable poor long-term retention of acquired skills and knowledge [25,32,33]. Taking into account available resources and infrastructures in post-conflict regions and identifying essential improvements for the CME programme is essential for effective improvement of health services [16,18].

There are two limitations that stand out for using qualitative methods for evaluation of CME for post-conflict zones. First, the programme evaluators need specialized training in qualitative methods for rigorous and valid results. Secondly, though qualitative methods identify deficiencies in post-conflict zones, these methods cannot quantitatively measure the scope of the lack of equipment, supplies, support personnel and facilities in post-conflict zones. A follow-up study is required to determine the magnitude of these deficiencies.

## Conclusions

CME is one of the most effective ways of rebuilding the health care system in post-conflict zones. The optimal way for informing and directing the adaptation of CME for these zones is through qualitative evaluations. The qualitative evaluation identifies both successes and obstacles in CME in post-conflict zones that would otherwise be missed with traditional quantitative survey methods. The study highlights the fact that the health care human resources training should be closely linked to appropriate technologies, supplies, facilities and human resources available in post-conflict zones. A qualitative research approach most effectively identifies these limitations and can directly inform the optimal adjustments for effective CME planning in these zones. These qualitative methods also provide targets for the donation of equipment, supplies and renovations that are needed in these zones.

## Abbreviations

CME: continuing medical education; FGD: focus group discussion; IDI: in-depth interview; NK: Nagorno Karabagh.

## Competing interests

The funds for evaluation of the programme were provided by Armenian American Health Professionals’ Organization to the Fund for Armenian Relief. The first and second authors are employees of the Fund for Armenian Relief, and the third author is on the board of the Armenian American Health Professionals Organization.

## Authors’ contributions

AB designed the study, performed data collection and analysis of collected data, HS participated in design of study and analysis of data, KH participated in design of study and helped to draft the manuscripts, BC helped in analysis of data and drafting the manuscript. All authors read and approved the final manuscript.
